# Novel Dental Low-Shrinkage-Stress Composite with Antibacterial Dimethylaminododecyl Methacrylate Monomer

**DOI:** 10.3390/jfb14070335

**Published:** 2023-06-25

**Authors:** Abdullah Alhussein, Rashed Alsahafi, Xiaohong Wang, Heba Mitwalli, Hanan Filemban, Gary D. Hack, Thomas W. Oates, Jirun Sun, Michael D. Weir, Hockin H. K. Xu

**Affiliations:** 1 PhD Program in Dental Biomedical Sciences, University of Maryland School of Dentistry, Baltimore, MD 21201, USA; aalhussein@ksu.edu.sa; 2 Department of Restorative Dental Sciences, College of Dentistry, King Saud University, Riyadh 11451, Saudi Arabia; 3 Department of Restorative Dental Sciences, Umm Al-Qura University, College of Dentistry, Makkah 24211, Saudi Arabia; 4 American Dental Association Science and Research Institute, LLC., Gaithersburg, MD 20899, USA; 5 Department of Operative Dentistry, Faculty of Dentistry, King Abdulaziz University, Jeddah 21589, Saudi Arabia; 6 Biomaterials & Tissue Engineering Division, Department of Advanced Oral Sciences and Therapeutics, University Maryland School of Dentistry, Baltimore, MD 21201, USA; 7 The Forsyth Institute, Harvard School of Dental Medicine Affiliate, Cambridge, MA 02142, USA; 8 Center for Stem Cell Biology & Regenerative Medicine, University of Maryland School of Medicine, Baltimore, MD 21201, USA; 9 Marlene and Stewart Greenebaum Cancer Center, University of Maryland School of Medicine, Baltimore, MD 21201, USA

**Keywords:** oral biofilms, caries, antibacterial, bio-interactive, resin composite, low polymerization stress

## Abstract

Objectives: Current dental resins exhibit polymerization shrinkage causing microleakage, which has the potential to cause recurrent caries. Our objectives were to create and characterize low-shrinkage-stress (LSS) composites with dimethylaminododecyl methacrylate (DMADDM) as an antibacterial agent to combat recurrent caries. Methods: Triethylene glycol divinylbenzyl ether and urethane dimethacrylate were used to reduce shrinkage stress. DMADDM was incorporated at different mass fractions (0%, 1.5%, 3%, and 5%). Flexural strength, elastic modulus, degree of conversion, polymerization stress, and antimicrobial activity were assessed. Results: The composite with 5% DMADDM demonstrated higher flexural strength than the commercial group (*p* < 0.05). The addition of DMADDM in BisGMA-TEGDMA resin and LSS resin achieved clinically acceptable degrees of conversion. However, LSS composites exhibited much lower polymerization shrinkage stress than BisGMA-TEGDMA composite groups (*p* < 0.05). The addition of 3% and 5% DMADDM showed a 6-log reduction in *Streptococcus mutans (S. mutans)* biofilm CFUs compared to commercial control (*p* < 0.001). Biofilm biomass and lactic acid were also substantially decreased via DMADDM (*p* < 0.05). Conclusions: The novel LSS dental composite containing 3% DMADDM demonstrated potent antibacterial action against *S. mutans* biofilms and much lower polymerization shrinkage-stress, while maintaining excellent mechanical characteristics. The new composite is promising for dental applications to prevent secondary caries and increase restoration longevity.

## 1. Introduction

Currently, methacrylate-based composites are the most widely used restorative materials in dentistry [1]. These materials have several advantages, making them the materials of choice for dental practitioners, such as esthetics, improved wear resistance, and the capacity to bond to dentin and enamel [1]. Failure of resin composite restorations still occur, however, with the survival rate ranging between 5 and 10 years [2]. The major etiological factors for resin composite failures are recurrent caries and tooth fractures [3]. These failures could be due lack of bioactivity and the development of polymerization shrinkage stresses, respectively, at the tooth-restoration interface [3]. Polymerization shrinkage stress initiates during polymerization [4], wherein the composite shrinks and loses the ability to flow, leading to residual shrinkage stresses due to the bonding restraint between the composite and tooth structures [5,6,7]. As a result, the polymerization shrinkage stress may lead to debonding at tooth-restoration interfaces, resulting in marginal gap formation and micro-cracking [6,7,8,9]. The marginal gaps at the tooth-restoration interfaces may lead to recurrent caries, especially with the lack of bioactivity in resin-based dental materials [10].

Several methods have been investigated to reduce shrinkage stresses [11,12]. These strategies include changing the resin matrix’s chemistry by the use of resin systems with a unique polymerization behavior, such as epoxy resins [13], siloranes [14], and step-growth thiolene resins [15]. Furthermore, the use of epoxy oligomers or polymeric nanogels could be beneficial in reducing shrinkage stress [11,12]. Recently, a low-shrinkage-stress (LSS) resin was developed using urethane dimethacrylate (UDMA) as a base monomer and ether-based triethylene glycol divinylbenzyl ether (TEG-DVBE) as a diluent, which resulted in a lower polymerization rate by delaying the gel point phase [16]. As a result, this approach allows more time for the resin composite to reach the gel point, providing stress relaxation and preventing accumulation of excessive contraction stresses [17]. Additionally, UDMA can increase the resistance of the resin-based material to salivary hydrolysis, which can diminish material degradation [17]. TEG-DVBE can also resist esterase degradation and hydrolytic challenges [17]. In a recent study, the UDMA/TEG-DVBE system was incorporated into a dental adhesive, showing decreased water sorption and solubility [18]. In addition, the UDMA/TEG-DVBE dental adhesive formed more resin tags and thicker hybrid layers than commercially available adhesives [18].

Another approach to improving the longevity of resin-based materials involves using bioactive agents within the materials’ formulations [19,20,21]. Among several antibacterial materials, quaternary ammonium methacrylates (QAMs) had a potent antibacterial effect against dental biofilm and promising results for potential clinical applications [19]. Among the monomers derived from QAMs, DMADDM with 12-unit -CH_2_- chain length and dimethylaminohexadecyl methacrylate (DMAHDM) with 16-unit -CH_2_- chain length stood out [20]. These monomers showed a strong antibacterial effect against dental biofilm [20,21]. However, incorporating DMAHDM in resin composites showed inconsistent antibacterial properties in previous studies [22,23,24]. In addition, mixing DMAHDM in resins is a challenge due to the high viscosity of DMAHDM. In contrast, DMADDM can be more readily mixed with other resin monomers, thus reducing the processing and handling challenges. The DMADDM monomer can induce bacterial death by interacting with the positive charge quaternary amine N^+^ and the negative charge cell membrane, providing a contact-killing antimicrobial effect [25]. There were several attempts to incorporate DMADDM in dental materials [26,27,28]. In a previous study, DMADDM was incorporated in acrylic denture base material, which inhibited multi-species biofilm consisting of *Candida albicans, S. mutans*, *Streptococcus sanguinis*, and *Actinomyces naeslundii* [28]. Furthermore, the biofilm biomass was significantly reduced by incorporating 3.3% of DAMDDM [28]. In addition, there was an attempt to use DMADDM as a coating agent for dental implants, which reduced the metabolic activity and biofilm growth of multi-species biofilms [29]. Furthermore, they found that DMADDM-modified titanium implants could inhibit the biofilm growth of *Neisseria* and *Actinomyces* spp. [29]. Recently, the incorporation of DMADDM in resins showed a strong antibacterial effect against dental biofilms [26,27]. However, a literature search revealed no report on incorporating DMADDM in LSS resins.

Improving the longevity of dental restorations is a main goal in the dental material field; developing a bioactive LSS resin composite could enhance the marginal seal and prevent secondary caries [30,31,32]. Previously, it was shown that the sealing ability was enhanced with a low shrinkage-stress composite, which may inhibit secondary caries [31]. Other studies investigated the incorporation of DMADDM with resin-based material, showing promising antibacterial activity without compromising mechanical properties [27,33]. However, no report has investigated the antibacterial and mechanical characteristics of incorporating DMADDM with a LSS resin composite.

Therefore, this study aimed to elucidate the effect of DMADDM concentration dependence on mechanical properties, antibacterial response, and polymerization rate for LSS resin composite restorations. It was hypothesized that: (1) mechanical properties of a novel LSS resin composite would not be compromised with the incorporation of different mass fractions of DMADDM; (2) the novel composite with the incorporation of DMADDM would reduce biofilm viability, lactic acid production, and metabolic activity with increasing the DMADDM mass fraction; and (3) a reduction in the polymerization shrinkage stress would occur by utilizing the LSS composite.

## 2. Materials and Methods

### 2.1. Formation of Composites Containing Different Mass Fractions of DMADDM

The low-shrinkage-stress (LSS) resin was formulated using 55.8% UDMA (Esstech, Essington, PA, USA) and 44.2% TEG-DVBE (mass %), following previous studies [30,34]. This resin was designated as “UV”. The 0.2% camphorquinone (CQ, Millipore Sigma, Burlington, MA, USA) and 0.8% of 4-N, N-dimethylaminobenzoate (4EDMAB; Millipore Sigma, Burlington, MA, USA) were added as photoinitiators.

**Figure 1:**
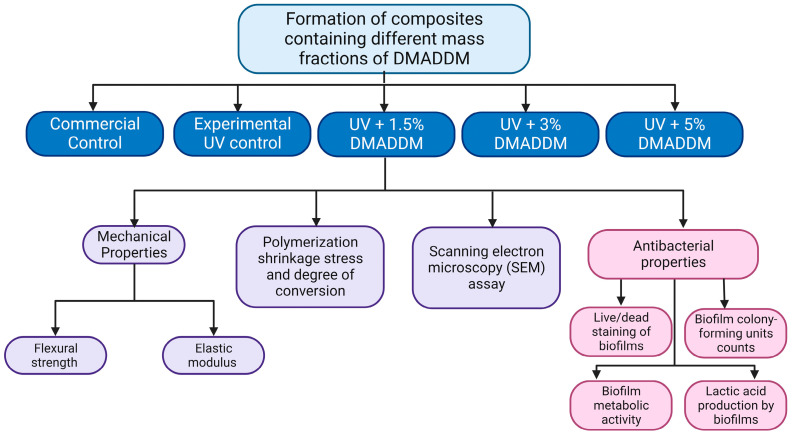
Schematic of the study design. The figure shows the optimization of antibacterial composites that incorporate varying mass fractions of DMADDM. Furthermore, the figure highlights the evaluation of both microbiological analysis and mechanical properties. Created via BioRender.com on 4 June 2023.

DMADDM was synthesized via the addition reaction of tertiary amines with organo-halides [35,36]. To synthesize DMADDM with CL of 12, 10 mmol of 2-(dimethylamino) ethyl methacrylate (DMAEMA, Aldrich, St. Louis, MO, USA), 10 mmol of 1-bromododecane (BDD) (TCI America, Portland, OR, USA), and 3 g of ethanol were added to a scintillation vial, which was capped and stirred at 70 °C for 24 h. Subsequently, ethanol was removed by evaporation [26]. The DMADDM was mixed with the UV resin to give final DMADDM concentrations in the resin composites of 0%, 1.5%, 3%, and 5% (weight %). The silanized barium boroaluminosilicate glass particles (d = 1.2 µm, Dentsply Sirona, Milford, DE, USA) were added at 70% mass fraction into the composite for mechanical reinforcement. The 70% glass filler level was chosen based on preliminary experiments to provide good handling properties (Table 1). In this study, Heliomolar (Ivoclar, Ontario, Canada) was used as a commercial control. It is a bioactive material that has the ability to release fluoride, according to previous studies [23,34]. The objective of this study was to investigate the antimicrobial efficacy and mechanical properties of the new formulations. To outline the experimental design process, a diagram depicting the procedures is shown in Figure 1.

The following were tested:

1. Heliomolar (designated as “Commercial Control”);
2. 30% UV + 0% DMADDM + 70% glass (designated as “Experimental UV control”);
3. 28.5% UV + 1.5% DMADDM + 70% glass (designated as “UV + 1.5% DMADDM”);
4. 27% UV + 3% DMADDM + 70% glass (designated as “UV + 3% DMADDM”);
5. 25% UV + 5% DMADDM + 70% glass (designated as “UV + 5% DMADDM”).

### 2.2. Mechanical Properties

For mechanical testing, a mold with specific dimensions of 2 × 2 × 25 mm^3^ was used to produce the composite bars [34]. Specimens were photopolymerized using a Labolight, DUO (GC, Tokyo, Japan) curing lamp for 1 min [34]. Flexural strength and elastic modulus (*n* = 6) were measured using a 3-point flexural test with a 10 mm span and a crosshead speed of 1 mm/min on a Universal Testing Machine (MTS, Insight 1, Cary, NC) [37].

### 2.3. Polymerization Shrinkage Stress and Degree of Conversion

**Figure 2:**
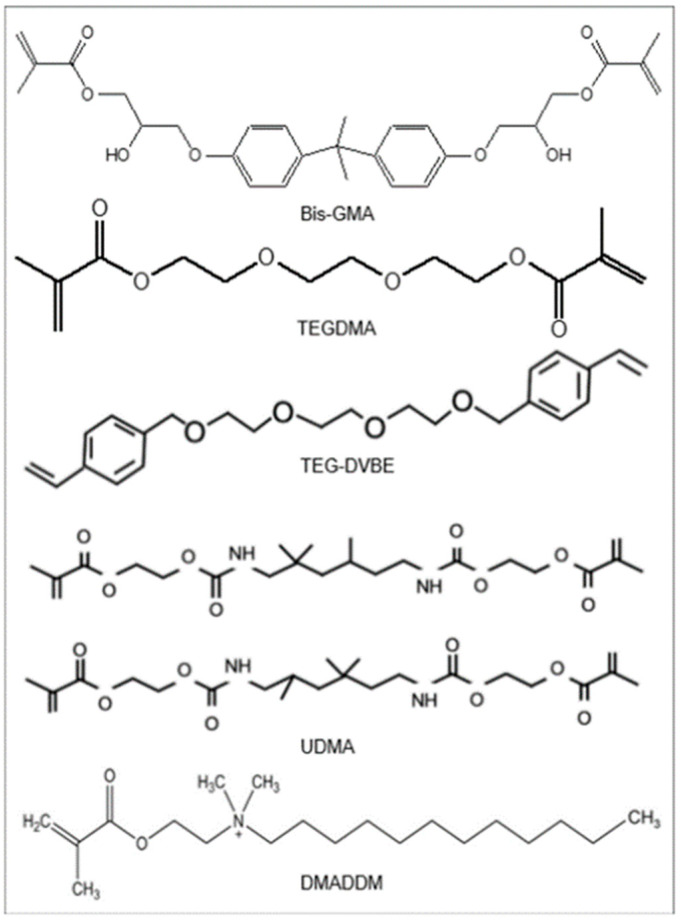
Chemical structure of monomers used in the resins.

In preliminary experiments, it was determined that Heliomolar had a low degree of conversion and therefore would not be a suitable comparison for degree of conversion and polymerization stress measurements [30]. Therefore, a traditional, high-DC resin composite was used as a comparison to assess the degree of conversion and polymerization stress of the LSS resin composite. The high DC composite consisted of bisphenol A glycidyl dimethacrylate (BisGMA, Esstech) and triethylene glycol dimethacrylate (TEGDMA, Esstech), mixed at a mass ratio of 1:1 (referred to as BT resin). The chemical structure of these monomers are shown in Figure 2.

The following groups were tested for polymerization stress and degree of conversion:

(1) 30% BT + 70% glass (designated as “Experimental BT control”);
(2) 28.5% BT + 1.5% DMADDM + 70% glass (designated as “BT + 1.5% DMADDM”);
(3) 27% BT + 3% DMADDM + 70% glass (designated as “BT + 3% DMADDM”);
(4) 25% BT + 5% DMADDM + 70% glass (designated as “BT + 5% DMADDM”);
(5) 30% UV + 70% glass (designated as “Experimental UV control”);
(6) 28.5% UV + 1.5% DMADDM + 70% glass (designated as “UV + 1.5% DMADDM”);
(7) 27% UV + 3% DMADDM + 70% glass (designated as “UV + 3% DMADDM”);
(8) 25% UV + 5% DMADDM + 70% glass (designated as “UV + 5% DMADDM”).

Polymerization shrinkage stress was investigated using a cantilever beam-based tensometer [38]. This device was linked to a near-infrared (NIR) spectrometer, allowing for the simultaneous monitoring of the ongoing conversion of double bonds, as previously described [39].

### 2.4. Streptococcus mutans (S. mutans) Biofilm Model

#### 2.4.1. Composite Samples for Biofilm Testing

Composite disks were fabricated (d = 9 mm, t = 2 mm) and each sample was photopolymerized for 60 s per side and stored at 37 °C for 24 h [34]. Samples were placed in distilled water and stirred for 1 h to facilitate removal of the uncured monomer [40]. Ethylene oxide (Anprolene AN 74i, Andersen, Haw River, NC, USA) was used to sterilize the resin composite disks (*n* = 6). Samples were de-gassed for seven days, following the manufacturer’s instructions.

#### 2.4.2. Bacteria Inoculation and Biofilm Formation

Bacterial species were approved for use by the University of Maryland Baltimore Institutional Review Board. As a result of its association with dental caries, *S. mutans* (UA159) was chosen as the bacterial species for this study [41]. *S. mutans* was cultured overnight (16–18 h) in brain heart infusion (BHI) broth (Sigma-Aldrich, St. Louis, MO, USA) at 37 °C with 5% CO_2_ [34]. A spectrophotometer (Genesys 10S, Thermo Scientific, Waltham, MA) was used to adjust the inoculum to 10^7^ colony-forming unit counts CFU/mL, based on the standard curve of OD_600_ nm versus the CFU/mL graph [42]. Each composite disk was set in the well of 24-well plates, covered with 1.5 mL BHI culture medium supplemented with 2% sucrose, and incubated for 24 h. Subsequently, the composite disks were transferred to 24-well plates, covered with 1.5 mL of fresh medium with sucrose, and incubated for another 24 h. According to a previous study, incubation for 48 h was adequate to form extensive biofilms on dental composites [43].

#### 2.4.3. Live/Dead Staining of Biofilms

The biofilm-covered composite disks were washed with phosphate buffered saline (PBS) to remove planktonic bacteria. The resin composite samples were stained with the BacLight live/dead kit (Molecular Probes, Eugene, OR, USA). Each disk was incubated with 2.5 µM SYTO 9 and 2.5 µM propidium iodide for 15 min. The presence of live bacteria was stained with SYTO9 and emitted green fluorescence. Bacteria with damaged membranes were stained with propidium iodide and emitted red fluorescence. A fluorescence microscope (Eclipse TE2000-S, Nikon, Melville, NY, USA) was used to assess the biofilms on the disks [44].

#### 2.4.4. Biofilm Colony-Forming Units Counts

Biofilm-attached disks were moved to a dish containing PBS and biofilms were harvested by a combination of scraping and sonication/vortexing [30]. The bacterial suspensions were serially diluted (10^1^–10^6^-fold) and spread on BHI agar plates. Agar plates were incubated for 48 h at 37 °C and 5% CO_2_, the colony number was counted, and the biofilm colony-forming units (CFU) counts were determined [43]. The CFU experiment was performed in triplicate.

#### 2.4.5. Biofilm Metabolic Activity

The biofilm metabolic activity was measured by a 3-[4,5- dimethylthiazol-2-yl]-2,5- diphenyltetrazolium bromide (MTT) assay [45]. Composite samples with attached biofilms for 48 h were transferred to a 24-well plate containing 1 mL of MTT dye (0.5 mg/mL MTT in PBS) and incubated at 37 °C in 5% CO_2_ for 1 h [45]. Subsequently, each disk was moved into a new 24-well plate containing 1 mL DMSO in each well and incubated at room temperature in the dark for 20 min. For the absorbance calculation, 200 µL of the DMSO solution was added to a 96-well plate, and the absorbance was measured at 540 nm [45]. Higher absorbance values denote an increased biofilm metabolic activity [45]. The metabolic activity experiment was performed in triplicate.

#### 2.4.6. Lactic Acid Production by Biofilms

The resin composite samples with attached biofilms for 48 h were transferred to 24-well plates filled with buffered peptone water (BPW, Aldrich, St. Louis, MO, USA), supplemented with 0.2% sucrose and incubated at 37 °C in 5% CO_2_ for 3 h [35]. The lactate dehydrogenase enzymatic was used to determine lactate concentrations in BPW thru measuring optical density at 340 nm using a microplate reader (Spectra-Max M5) as previously described [40]. The lactic acid production experiment was performed in triplicate.

#### 2.4.7. Scanning Electron Microscopy (SEM)

The composites were investigated in SEM (Quanta 200, FEI Company, Hillsboro, OR, USA). The specimens were polished with 4000 grit sandpaper, followed by sputter-coating with gold (*n* = 6).

For SEM examination of biofilms, the biofilms on the composite disks (*n* = 6) at 48 h were cleaned with PBS and then soaked in 1% glutaraldehyde at 4 °C overnight. Then they were washed with PBS and subjected to dehydration using a sequence of ethanol solutions. They were then washed with hexamethyldisilazane and allowed to air-dry overnight. The samples were sputter-coated with platinum and examined in SEM.

### 2.5. Statistical Analysis

Statistical analyses were performed using Sigma Plot (SYSTAT, Chicago, IL, USA). One-way analyses of variance (ANOVA) and Tukey’s comparison tests were performed to detect the significant differences between groups. Results were considered statistically significant at a *p*-value of less than 0.05.

## 3. Results

### 3.1. Mechanical Properties

**Figure 3:**
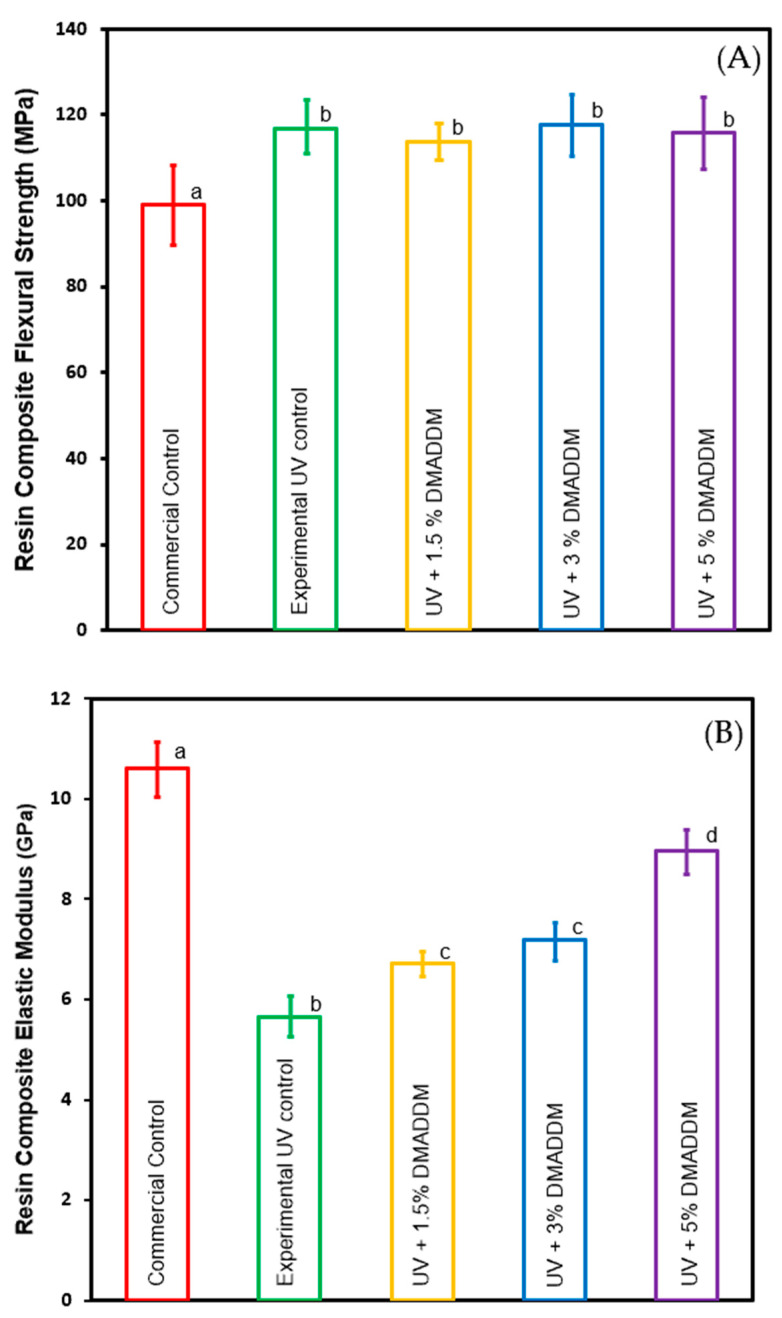
Mechanical properties of tested composites (**A**) Flexural strength and (**B**) elastic modulus (mean ± sd; n =6). Incorporating 5% DMADDM in a LSS resin composite significantly increased flexural strength compared to commercial group (*p* < 0.05). The increase of DMADDM concentration by up to 5% increased the elastic of modules for UV resin compared to the experimental control (*p* < 0.05). LSS resin composite demonstrated significantly lower elastic of modules than commercial group (*p* < 0.05). Dissimilar letters indicate values that are significantly different from each other (*p* < 0.05).

Flexural strength and elastic modulus of the composites are shown in Figure 3 (mean ± sd; n =6). The addition of 1.5%, 3%, and 5% DMADDM in LSS resin composite resulted in an increase in flexural strength compared to the commercial group (*p* < 0.05). The experimental group and 3% DMADDM composite had the highest flexural strength among experimental groups and was significantly higher than the commercial control. However, they demonstrated similar values compared to other groups (*p* > 0.05). This suggests that incorporating up to 5% of DMADDM in LSS composite had minimal effect on the flexural strength.

The elastic modulus values of the commercial control group were significantly greater than all other experimental groups (*p* < 0.05). Increasing the DMADDM mass fraction in LSS resin composite increased the elastic modulus compared to the experimental group (*p* < 0.05).

### 3.2. Polymerization Shrinkage Stress and Degree of Conversion

**Figure 4:**
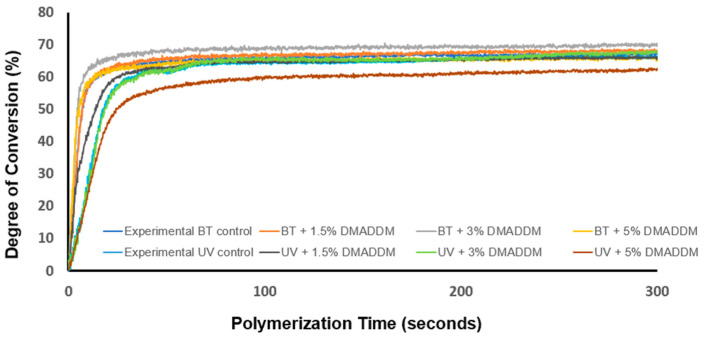
Degree of conversion as a function of polymerization time (*n* = 3). Incorporation of 5% DMADDM in LSS composite had the lowest polymerization rate. However, the degree of conversion for all groups is within the clinically acceptable level.

**Figure 5:**
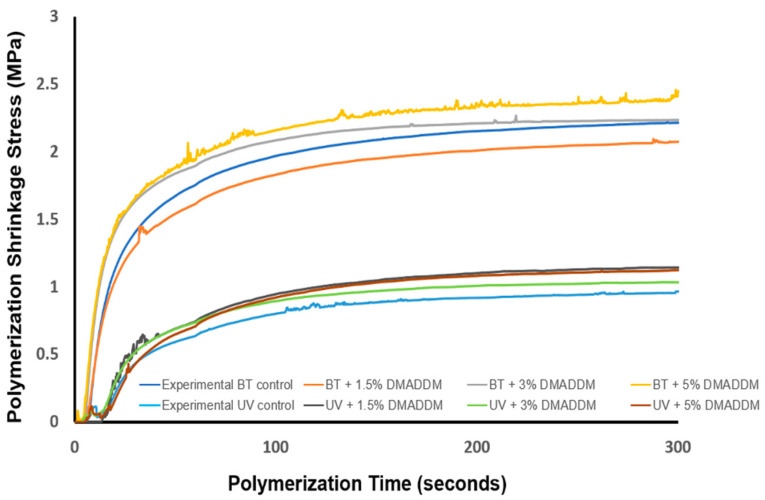
Polymerization shrinkage stress vs. polymerization time (*n* = 3). The LSS composite groups exhibited a postponement in the progression of polymerization shrinkage stress (*p* < 0.05). The composite containing BT and 5% DMADDM exhibited a polymerization shrinkage stress measuring 2.21 MPa. On the other hand, the UV composite with 5% DMADDM experienced a decrease in shrinkage stress, reaching 1.12 MPa.

Degree of conversion results are shown in Figure 4 (*n* = 3). The degree of conversion of all of the groups gradually increased with time until they reached the plateau. The result showed that UV resin takes a long time to reach its maximum compared to BT resin. The UV-containing composites with up to 3% DMADDM reaches a degree of conversion of approximately 67%. However, the increase of DMADDM to 5% in UV resin decreased the degree of conversion to 62%. Furthermore, our findings demonstrated that there is no significant difference in the degree of conversion after incorporating 3% DMADDM into UV resin and BT resin (*p* < 0.05). The polymerization shrinkage stress is plotted in Figure 5 (*n* = 3). The BT resin composite groups showed greater polymerization shrinkage stress than UV resin (*p* < 0.05). The addition of DMADDM up to 5% in UV-containing composites demonstrated comparable polymerization shrinkage stress (1.12 ± 0.06 MPa) to experimental UV resin (0.97 ± 0.05 MPa) (*p* > 0.05). In contrast, the incorporation of DMADDM up to 5% in BT resin resulted in increasing polymerization shrinkage stress (2.45 ± 0.14 MPa ) compared to experimental BT resin (2.22 ± 0.12 MPa ) (*p* > 0.05).

### 3.3. Live/dead Staining of S. mutans Biofilms

**Figure 6:**
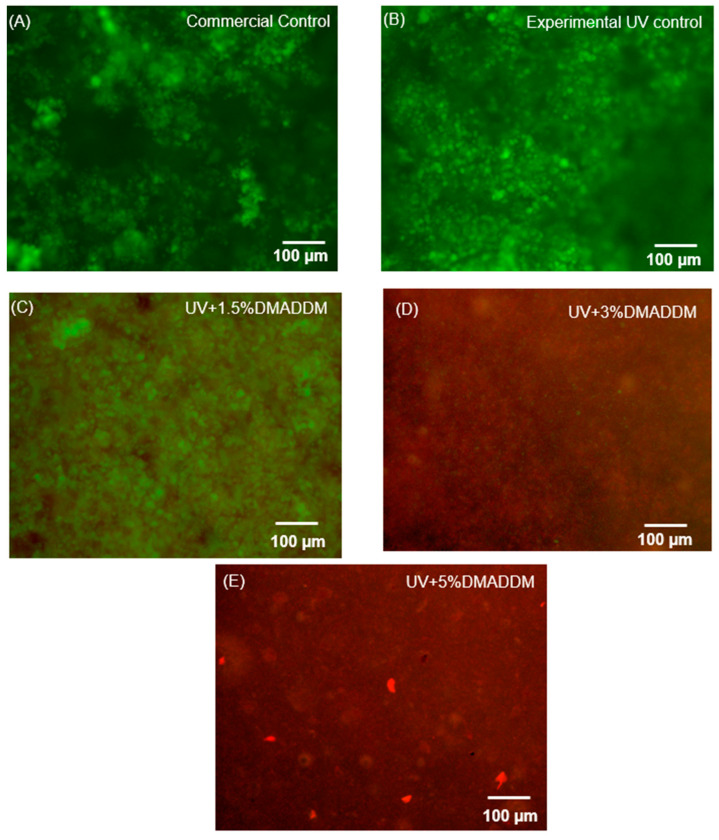
Descriptive live/dead staining images of biofilms on resin composite disks. (**A**) Commercial control. (**B**) Experimental control. (**C**) Composite with 1.5% DMADDM. (**D**) Composite with 3% DMADDM. (**E**) Composite with 5% DMADDM. Commercial and experimental control resin composite were covered by live bacteria (green stain). In contrast, the addition of DMADDM to LSS composite resulted in more dead bacteria (red stain).

Live/dead images of 48 h biofilms on the composites are illustrated in Figure 6. The surface of control groups had biofilms containing primarily live bacteria. The incorporation of DMADDM in the UV resin composite effectively inhibited biofilm growth, as demonstrated by the increased red staining observed with higher DMADDM mass fractions in the composite.

### 3.4. Biofilm Colony-Forming Units Counts

**Figure 7:**
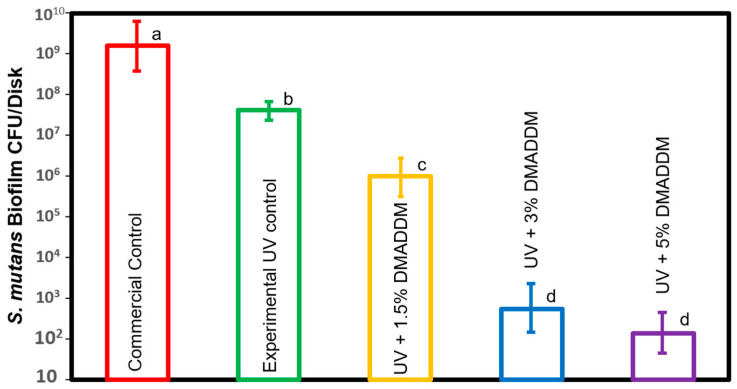
The colony-forming unit (CFU) counts of biofilms on composite disks (mean ± sd; n=6). Control groups had the highest biofilm growth. The increase in the DMADDM up to 5% significantly reduced the *S. mutans* biofilm growth compared to commercial control (*p* < 0.05). Adding 3% and 5% DMADDM into LSS resin composite significantly reduced the *S. mutans* biofilm growth by 6 orders of magnitude less than commercial control (*p* < 0.05). Dissimilar letters indicate values that are significantly different from each other (*p* < 0.05).

The CFU results of the *S. mutans* biofilm are shown in Figure 7 (mean ± sd; n=6). Incorporating 1.5% DMADDM in LSS resin composite significantly decreased the CFUs for *S. mutans* compared to control groups (*p* < 0.05). Conversely, increasing the DMADDM mass fraction to 3% and 5% in UV resin composite showed more reduction in the CFU count by 6–6.5 log compared to the commercial group (*p* < 0.05). However, there was no significant difference in reducing the CFU count among these groups (*p* > 0.05).

### 3.5. MTT Assay of Metabolic Activity of S. mutans Biofilms

**Figure 8:**
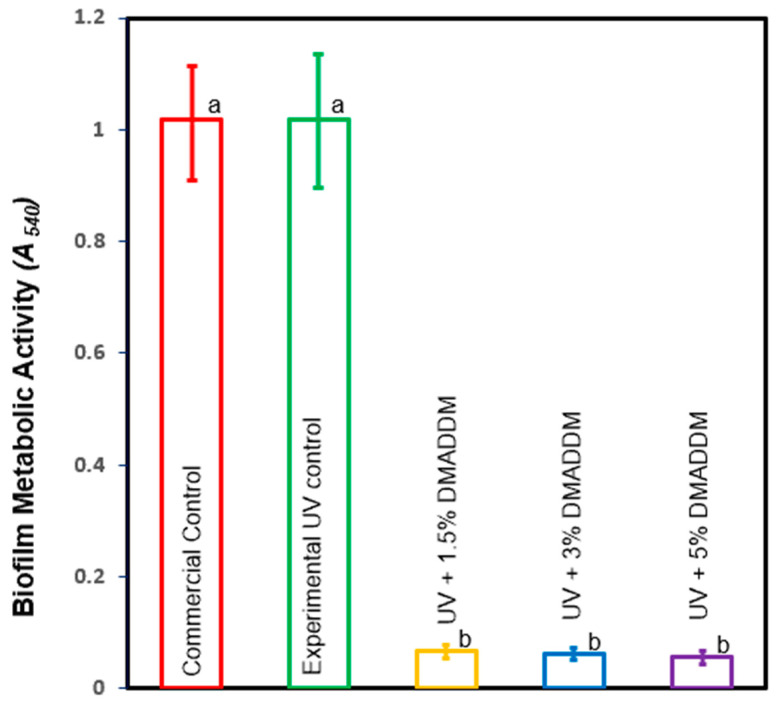
Metabolic activity of biofilm MTT (mean ± sd; n=6). Adding DMADDM to LSS resin composite reduced the metabolic activity of *S. mutans* compared with control groups (*p* < 0.05). Dissimilar letters indicate values that are significantly different from each other (*p* < 0.05).

The metabolic activity of 48 h biofilms on the composites are illustrated in Figure 8. Incorporating 1.5%, 3%, and 5% of DMADDM in UV resin significantly decreased the metabolic activities compared to the control groups (*p* < 0.05). However, there was no significant difference in the metabolic activities among these groups (*p* > 0.05).

### 3.6. Lactic Acid Production by S. mutans Biofilms

**Figure 9:**
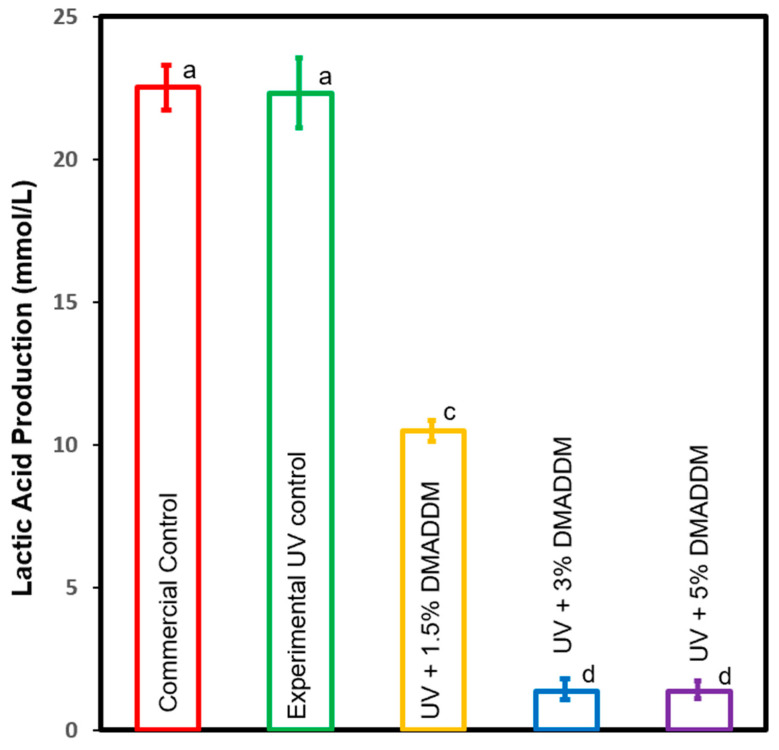
Lactic acid production by biofilms on resin composites (mean ± sd; n=6). The commercial and experimental control groups had the highest lactic acid concentration (*p* < 0.05). Incorporating 3% and 5% of DMADDM in LSS resin composite significantly decreased lactic acid production compared to other groups (*p* < 0.05). Dissimilar letters indicate values that are significantly different from each other (*p* < 0.05).

Lactic acid production of *S. mutans* biofilms adherent on the resin composites is depicted in Figure 9 $\left(\mathrm{mean}\;\pm \;\mathrm{sd};\;n=6\right)$. The control groups had the highest acid production (*p* < 0.5). Incorporating 3% and 5% DMADDM in UV resin composite resulted in a significant reduction of acid concentration compared to the commercial group (*p* < 0.05). Nevertheless, there was no significant difference in acid production among these groups (*p* > 0.05). The incorporation of 1.5% DMADDM showed less lactic acid than commercial groups (*p* < 0.05). However, it has higher lactic acid production than the 3% and 5% DMADDM groups (*p* < 0.05). This indicates that increasing DMADDM concentration to 3% and 5% resulted in the highest biofilm reduction.

### 3.7. SEM Examination of Composites and Biofilms

**Figure 10:**
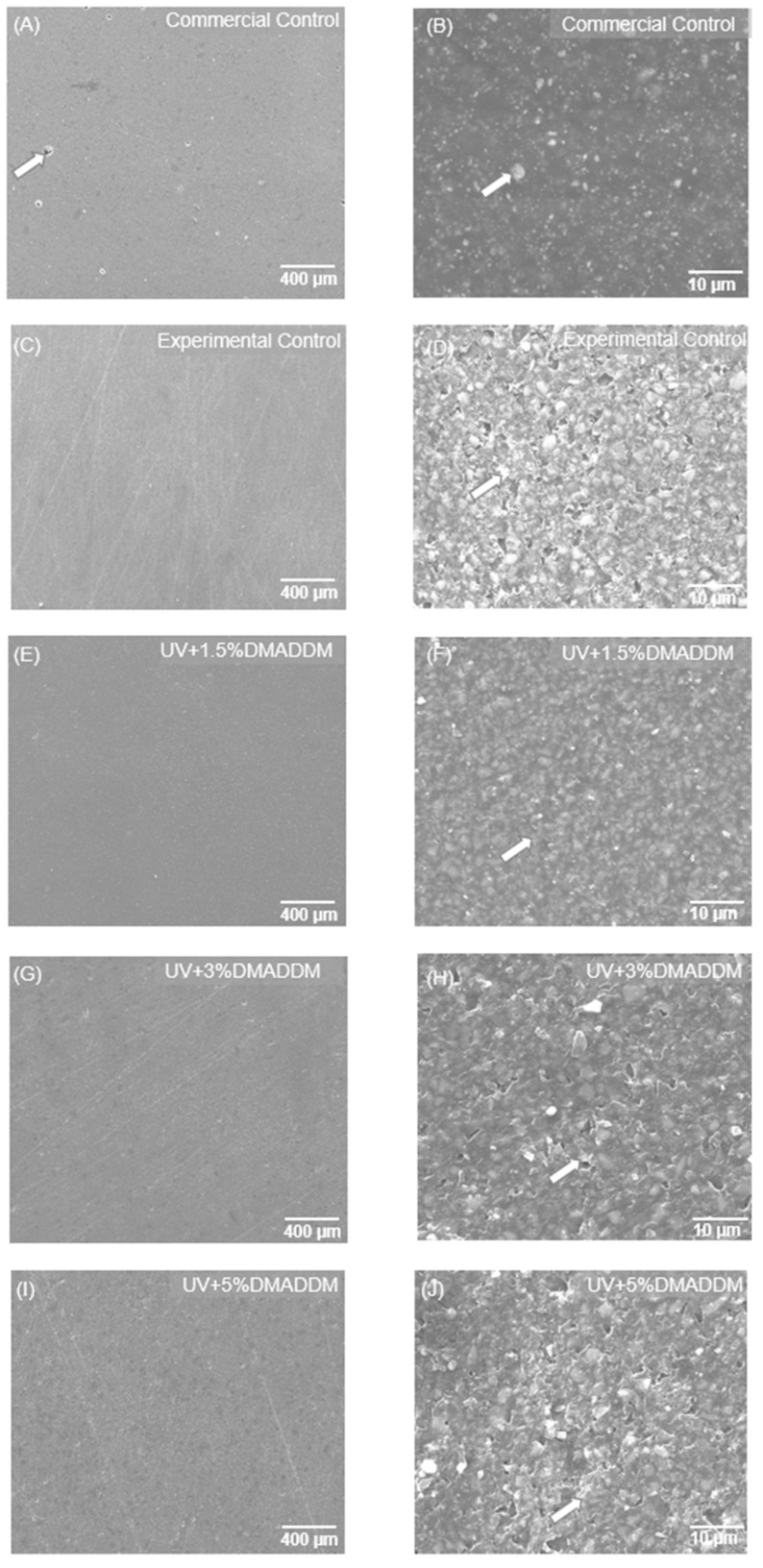
Representative SEM images of composites. (**A**,**B**) Commercial control composite. (**C**,**D**) Experimental control composite. (**E**,**F**) Composite with 1.5% DMADDM. (**G**,**H**) Composite with 3% DMADDM. (**I**,**J**) Composite with 5% DMADDM. All composites showed good filler particle distributions with a few small voids and absence of large agglomerates.

Representative SEM images of composites are shown in Figure 10 (*n* = 6). All experimental composites had a satisfactory distributions of filler particles with a few small voids, similar to the commercial control.

**Figure 11:**
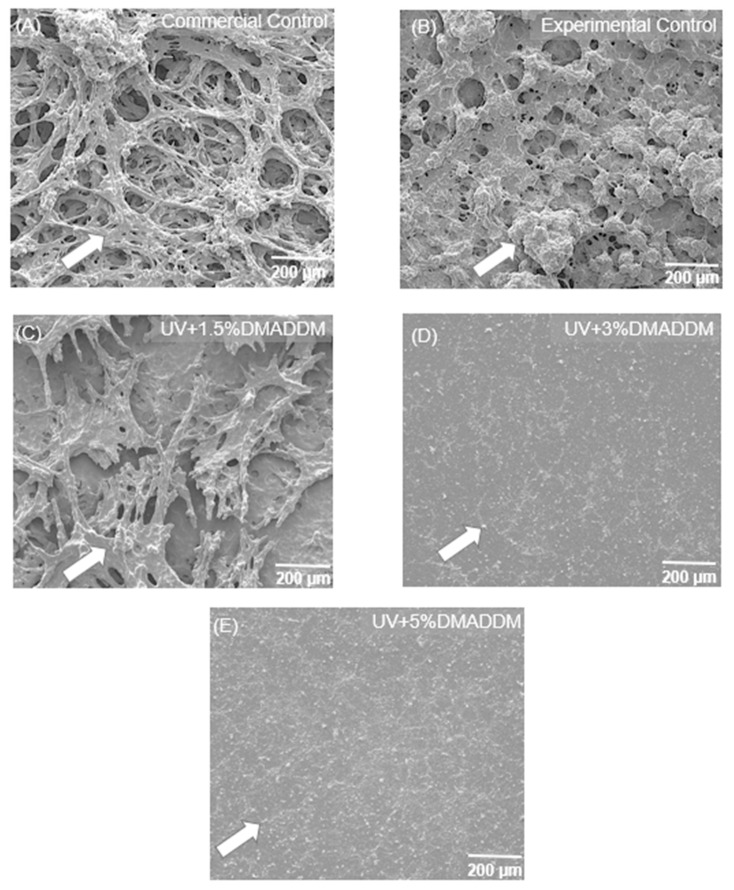
Representative SEM images of two-day biofilms on composites. (**A**) Commercial control. (**B**) Experimental control. (**C**) Composite with 1.5% DMADDM. (**D**) Composite with 3% DMADDM. (**E**) Composite with 5% DMADDM. The incorporation of 3% and 5% DMADDM into the LSS composite greatly reduced biofilm formation.

Representative SEM images of two-day *S. mutans* biofilms on composites are shown in Figure 11 (*n* = 6). The experimental control composite, the commercial control composite, and the 1.5% DMADDM composite exhibited substantial biofilm formation. In contrast, adding 3% and 5% DMADDM into the LSS composite led to minimal biofilm formation on the composites.

## 4. Discussion

This study explored the effect of the antimicrobial quaternary ammonium resin, DMADDM, in a (UDMA + TEG-DBVE) LSS composite. Incorporating of DMADDM in a LSS resin composite may reduce polymerization shrinkage stress and secondary caries, which could increase the clinical longevity of resin composite restoration [30,31,32]. Inhibition of *S. mutans* biofilm and reduction of polymerization shrinkage stress while maintaining excellent mechanical characteristics was accomplished, and the study hypotheses were proved. The adding of 3% DMADDM in low-shrinkage composite generated a 6-log reduction in *S. mutans* biofilm growth while maintaining clinically acceptable mechanical characteristics. Moreover, as the mass fraction of DMADDM increased, the production of lactic acid and metabolic activity within the biofilm of *S. mutans* decreased.

There were several successful endeavors to improve the mechanical characteristics and aesthetics of the composite resin restoration [46,47]. However, the most significant drawbacks of resin composite are polymerization shrinkage volume, and the resulting shrinkage stress that occurs during the polymerization process [6,7,8,9]. Polymerization shrinkage stress can potentially result in debonding at the interface between tooth structure and restoration, resulting in micro-cracking of the tooth structure and gap formation [3]. Therefore, developing a low shrinkage stress composite is highly desirable, which could help reduce stresses on the tooth structure and increase the survival rate of the restoration [31]. In a previous study, an attempt was made to minimize polymerization stress by using a thiolene system, which reduced stress development in a Bis-GMA/TEGDMA composite resin from 2.8 MPa to 0.30 MPa [48]. However, incorporating the thiolene system in resin composite compromised mechanical properties [48]. Another attempt to minimize polymerization stress by incorporating tricyclo decanedimethanol diacrylate (SR833s) and isobornyl acrylate (IBOA) as a diluent monomer, resulting in reducing stress development [49]. However, this approach compromised the degree of conversion [49]. In addition, a resin composite that is based on silorane was investigated in a long-term 12 month study, which found the marginal retention was not significantly improved compared with traditional methacrylate’s-based composite resins [50]. Recently, a LSS composite was developed utilizing TEGDVBE and urethane dimethacrylate monomers, which resulted in reduced polymerization stress, good mechanical strength, and a high degree of conversion [30]. Furthermore, several attempts were made to incorporate DMAHDM in LSS resin to develop a bioactive composite resin [34,43]. However, the DMAHDM has a long chain length, which increases the resin composite’s viscosity and could reduce the filler load and degree of conversion. Also, several articles showed inconsistent antibacterial results of incorporating DMAHDM in resin composite, which could be due to the long chain length of DMAHDM being present in conformations that result in a reduction in quaternary ammonium charge density at the surface [22,23,34]. However, no report has studied the impact of integrating different mass fractions of DMADDM in a LSS composite resin.

This study thoroughly investigated the effects of incorporating different amounts of DMADDM in a composite containing the low shrinkage stress resin TEGDVBE. The primary objective of this study was to determine the optimal concentration of DMADDM incorporated into the composite that maximized the antimicrobial response while maintaining clinically-desirable mechanical properties. The incorporation of DMADDM mass fractions up to 5% achieved higher flexural strength than the commercial control and was comparable to the experimental control. On the other hand, LSS composite resin with and without incorporation of DMADDM showed a lower modulus of elasticity than commercial control composite resin. It is likely that the higher elastic modulus for the commercial control composite was due to the differences in the resin matrix when compared to the experimental LSS composites. The LSS resin composite undergoes a slower or more regulated polymerization process to facilitate stress reduction, which could potentially result in a reduced elastic modulus. In addition, it is possible that the elastic modulus increase with increasing DMADDM concentration could be due to a higher viscosity of the resin matrix from DMADDM addition. The mechanical properties of composites can be adversely affected by the poor distribution of fillers and the presence of large voids or agglomerates. In the present study, SEM analysis of composites revealed a relatively uniform distribution of filler particles within the resin matrix for all DMADDM composites, with a few small voids and the absence of large agglomerates or large flaws. These features are similar to those of the commercial control. Maintaining an excellent mechanical property with enhancing the marginal seal of the dental restoration could help improves longevity [31,32]. Previous studies showed that polymerization shrinkage stress affects the sealing ability of resin composite [31]. Therefore, in this study, we developed a bioactive low-shrinkage-stress resin composite, which demonstrated a lower polymerization shrinkage stress than the BT composite, which may help achieve a better marginal seal. The incorporation of up to 5% DMADDM in UV resin composite reduced polymerization shrinkage stress at 1.12 MPa compared to 2.45 MPa for BT resin with 5% DMADDM. The difference in polymerization shrinkage stress may be due to resin monomers’ polymerization rate [51]. The UV resin takes a longer time to reach the gel point, allowing for stress relaxation and preventing excessive contraction stress development [17]. Creating novel bioactive LSS resin composites with antibacterial properties could help suppress acidogenic bacteria and provide a better marginal seal, which may help in improving the longevity of dental restoration. Maximizing the degree of conversion is essential in improving the durability and longevity of dental composites [52]. The degree of conversion of most dimethacrylate-based composites ranges from 55% to 75% [53]. In the current study, a comparatively high degree of conversion was achieved for both the UV resin composite, ranging from 62% to 67%, and the BT resin composite, ranging from 65% to 69%. Incorporating 5% DMADDM in UV and BT resin composite reduced the degree of conversion, which could be due to an increase in monomer viscosity with increasing DMADDM concentration. Future studies are necessary to fully understand the reaction kinetics for this new resin composite.

Dental plaque could affect the longevity of dental restoration, resulting in cariogenic bacterial attachment, hydrolytic enzyme production, material degradation, and recurrent caries [54,55,56]. Therefore, developing an innovative bioactive material with antibacterial properties could increase the durability of the composite restoration. In a previous study, chlorhexidine was incorporated as an antibacterial agent in a low-shrinkage composite, resulting in a short-term antibacterial effect [57]. Another attempt investigated incorporating antibacterial fillers silver and zinc oxide nanoparticles in dental materials [58]. These nanoparticle fillers tended to be lost from the dental material surface in a short period, which led to increased surface porosity and compromised mechanical properties [58]. A new approach was developed to overcome this critical drawback by using QAMs, which could be copolymerized with dental resins by covalent bonding, providing long-term antibacterial effects [59]. Incorporating QAMs in methacrylate-based materials showed a potent antimicrobial effect through a contact-killing mechanism [25]. The QAMs have positively charged quaternary amine, which can bind to the negatively charged bacterial cell membrane, changing the essential ion balance and cause cytoplasmic leakage, leading to disruption of the bacterial membrane [25]. Previous studies showed that increased chain length of QAMs resulted in more potent antibacterial properties due to increased hydrophobicity, which may improve their efficacy in disrupting the hydrophobic bacterial cell membrane [20,21]. Furthermore, the incorporation of DMADDM in resin-based material achieved a durable antibacterial activity even after six months of water aging, with sustained antibacterial function and mechanical properties [60]. In this study, the incorporation of DMADDM into a LSS resin composite showed a potent antibacterial effect against *S. mutans* biofilm. The incorporation of 3% and 5% DMADDM into LSS resin composite significantly reduced the CFU count by 6 and 6.5 logs, respectively, compared to the commercial control (*p* < 0.05). In addition, our results showed that incorporating 3% and 5% of DMADDM in LSS resin composite achieved more than 85% reduction in lactic acid production and metabolic activities compared to the commercial control. Furthermore, live/dead staining images of *S. mutans* biofilms confirm the presence of living bacteria on the surface of experimental and commercial control groups. On the other hand, increasing DMADDM concentration in LSS composites reduces the live bacteria present on the surface. Moreover, SEM examination of *S. mutans* biofilms showed substantial biofilm growth on the experimental control composite, the commercial control composite, and the 1.5% DMADDM composite. In contrast, adding 3% and 5% DMADDM led to minimal biofilm growth on the LSS composite. However, our results observed no significant difference in antibacterial effect when incorporating 3% or 5% DMADDM against *S. mutans* biofilms (*p* > 0.05).

Regarding biocompatibility, a previous study showed that the incorporation of 10% DMADDM into a Scotchbond multi-purpose adhesive achieved excellent cell viability that matched that of the control group [21]. Another in vivo investigation showed that the incorporation of 5% DMADDM into the adhesive and composite showed biocompatibility matching the control group [61]. Moreover, another study investigated the cell viability of human gingival fibroblast cells of uncured traditional monomers and the new LSS monomers [30]. That study found no significant differences in cell viability and cytotoxicity between the LSS monomers and the control monomers already used clinically [30]. Therefore, the DMADDM composite has the capability to inhibit bacterial biofilms without compromising biocompatibility.

In summary, the present study systematically examined the effect of incorporating different concentrations of DMADDM on the mechanical and antibacterial properties of LSS resin composite for the first time. Incorporating 3% DMADDM into the LSS composite resulted in strong antibacterial properties without compromising mechanical properties, with comparable results to the LSS resin composite with 5% DMADDM. The incorporation of 3% DMADDM reduced biofilm CFU counts by six orders of magnitude compared to commercial resin composite. Therefore, developing an antibacterial LSS resin composite is a promising approach to overcoming secondary caries and increasing the clinical longevity of the resin composite restoration.

Additional studies are needed to explore the antibacterial property of LSS resin composite on multi-species biofilms that are more clinically relevant. Also, further studies are required to explore the long-term effect of DMADDM on the antibacterial and mechanical characteristics of LSS resin composites. In addition, future research is needed to investigate the thermal stability and long-term degradation behavior of the novel LSS composites.

## 5. Conclusions

This study developed a novel antibacterial low-shrinkage-resin composite. Incorporating 3% DMADDM into the resin composite provided a strong antibacterial effect against *S. mutans* biofilms which is commonly associated with secondary caries, without compromising the mechanical properties. Incorporating 3% DMADDM into this novel dental composite reduced the polymerization stress without negatively impacting the degree of conversion. In addition, this formulation achieved a 6-log reduction in biofilm CFUs with a significant decrease in lactic acid production and metabolic activity. These results indicate that a dental resin composite system composed of the low shrinkage stress resin TEGDVBE combined with the antimicrobial resin DMADDM may be a promising bioactive dental restoration that could reduce the onset of secondary caries.

## Figures and Tables

**Table 1:** Compositions of LSS composites.

Composition	Experimental UV Control (Weight %)	UV + 1.5% DMADDM (Weight %)	UV + 3% DMADDM (Weight %)	UV + 5% DMADDM (Weight %)
Urethane dimethacrylate (UDMA)	16.57%	15.74%	14.92%	13.81%
Triethylene glycol divinylbenzyl ether (TEG-DVBE)	13.13%	12.46%	11.82%	10.94%
Camphorquinone	0.06%	0.06%	0.06%	0.06%
4-N, N-dimethylaminobenzoate	0.24%	0.24%	0.24%	0.24%
Dimethylaminododecyl methacrylate (DMADDM)	0%	1.5%	3%	5%
Silanized barium boroaluminosilicate glass particles	70%	70%	70%	70%

## Data Availability

Not applicable.
